# Homeostatic structural plasticity increases the efficiency of small-world networks

**DOI:** 10.3389/fnsyn.2014.00007

**Published:** 2014-04-01

**Authors:** Markus Butz, Ines D. Steenbuck, Arjen van Ooyen

**Affiliations:** ^1^Simulation Lab Neuroscience, Bernstein Facility for Simulation and Database Technology, Institute for Advanced Simulation, Jülich Aachen Research Alliance, Forschungszentrum JülichJülich, Germany; ^2^Student of the Medical Faculty, University of FreiburgFreiburg, Germany; ^3^Department of Integrative Neurophysiology, VU University AmsterdamAmsterdam, Netherlands

**Keywords:** topology, development, neuronal network model, structural synaptic plasticity, homeostasis, small-world network, efficiency

## Abstract

In networks with small-world topology, which are characterized by a high clustering coefficient and a short characteristic path length, information can be transmitted efficiently and at relatively low costs. The brain is composed of small-world networks, and evolution may have optimized brain connectivity for efficient information processing. Despite many studies on the impact of topology on information processing in neuronal networks, little is known about the development of network topology and the emergence of efficient small-world networks. We investigated how a simple growth process that favors short-range connections over long-range connections in combination with a synapse formation rule that generates homeostasis in post-synaptic firing rates shapes neuronal network topology. Interestingly, we found that small-world networks benefited from homeostasis by an increase in efficiency, defined as the averaged inverse of the shortest paths through the network. Efficiency particularly increased as small-world networks approached the desired level of electrical activity. Ultimately, homeostatic small-world networks became almost as efficient as random networks. The increase in efficiency was caused by the emergent property of the homeostatic growth process that neurons started forming more long-range connections, albeit at a low rate, when their electrical activity was close to the homeostatic set-point. Although global network topology continued to change when neuronal activities were around the homeostatic equilibrium, the small-world property of the network was maintained over the entire course of development. Our results may help understand how complex systems such as the brain could set up an efficient network topology in a self-organizing manner. Insights from our work may also lead to novel techniques for constructing large-scale neuronal networks by self-organization.

## 1. Introduction

The synaptic wiring of cortical networks is key to the functionality of the brain and a precondition for all cognitive behavior (Park and Friston, 2013). How are synaptic connections set up between the brain's billions of neurons so that cost efficiency (Latora and Marchiori, 2001), for example in terms of wiring length or energy consumption associated with information transmission, is maximized? Due to the presence of long-range connectivity, the brain can be regarded as a highly efficient graph, in the sense that information has to pass only a few intermediate neurons (“nodes”) to travel across the whole brain (Kaiser and Hilgetag, 2006). At the same time, brain connectivity is local and clustered (Hilgetag and Kaiser, 2004), making networks less vulnerable because of the many alternative routes that exist between two nodes. Network topology that combines these two properties, long-range connectivity and high clustering, is called small-world (Watts and Strogatz, 1998). Small-world networks have the advantages of local connectivity combined with a high efficiency brought about by a small number of long-range connections (Watts and Strogatz, 1998). The brain seems to be optimized for maximizing cost efficiency of parallel information processing (Achard and Bullmore, 2007) by widely adopting small-world topology (Sporns and Zwi, 2004; Bassett and Bullmore, 2006). Already in the infant brain, connectivity has small-world characteristics (Fransson et al., 2011). A recent fMRI study reported an increase in small-worldness of brain networks in the first 2 years of life that goes along with a growing number of long-distance connections and therefore an increase in global efficiency (Gao et al., 2011). So far, analysis of topology has focused on neuronal networks with static connectivity, in which plasticity arises from changes in the strength of existing synapses rather than from the rewiring of connectivity. However, particularly during development but also in adulthood, connectivity is not fixed (Butz et al., 2009b), and synapse formation goes along with massive synapse deletion and reorganization of connectivity (Missler et al., 1993; Siegel and Lohmann, 2013). This observation triggered the question of this study: how does network topology develop during ontogeny when synapse formation and pruning cause a constant rewiring of network connectivity?

Various explanations have been proposed to account for the development of synaptic connectivity. For example, axon guidance molecules may form the basis of a genetically-encoded developmental scheme (Yamamoto et al., 2002; Borisyuk et al., 2008). Target neurons may secret signaling molecules that can attract or repel axons. Axons can then follow or move away from the concentration gradient. This form of chemotaxis is usually discussed in the context of the formation of global connectivity. However, for the formation of local connectivity chemical cues are less suitable, since they fail to establish stable gradients over very short distances, below 0.7 mm (Kaiser et al., 2009). Synaptic adhesion molecules (e.g., neuroligins) were proposed as molecular cues for local synapse formation (Scheiffele et al., 2000; Stan et al., 2010). In addition, mechanical forces in the tissue could influence neurite outgrowth (Franze, 2013). Alternatively, the formation of local connectivity may basically be random (Braitenberg and Schüz, 1998), just depending on the accidental overlap of axons and dendrites (Binzegger et al., 2004; van Pelt and van Ooyen, 2013; McAssey et al., 2014; van Ooyen et al., 2014). With random synapse formation, the chance of forming connections decreases with increasing distance between neurons (Kaiser et al., 2009).

Although random overlap of axons and dendrites may explain emerging connectivity, it does not account for the actual driving forces underlying neurite outgrowth. There is ample evidence that electrical activity shapes neuronal morphology (Dalva et al., 1994; Wong and Ghosh, 2002; Uesaka et al., 2005; Butz et al., 2009b) and network formation (Ko et al., 2013). Electrical activity influences neurite outgrowth and retraction (McKinney et al., 1999a; Konur and Ghosh, 2005; Lohmann and Wong, 2005; Tailby et al., 2005; Hutchins and Kalil, 2008), as well as the formation and deletion of axonal boutons and dendritic spines (McKinney et al., 1999b; Groc et al., 2002; Jourdain et al., 2003; Kirov et al., 2004; Hofer et al., 2009). Experimental findings further suggest that neuronal morphogenesis is driven by the need of neurons to establish and maintain a homeostatic equilibrium of their average electrical activity (Kirov et al., 2004; Keck et al., 2008, 2013). Restoration of neuronal firing rate after a change in neuronal input has been found experimentally after, for example, focal retinal lesions (Hengen et al., 2013). Based on these experimental findings, we postulated that whenever during development (van Ooyen and van Pelt, 1994; Van Ooyen et al., 1995; Tetzlaff et al., 2010) or in the mature brain (Butz et al., 2008; Butz and van Ooyen, 2013) a neuron senses a deviation of its electrical activity from a homeostatic set-point, it will initiate changes in its morphology that increase the chance of synapse formation or break existing connections so that its firing rate may be restored. Here we investigate what the impact is of neurons regulating their electrical activities homeostatically on network formation and emerging network topologies.

In order to study the impact of electrical activity on emerging network topology, we used our recent Model of Structural Plasticity (MSP) (Butz and van Ooyen, 2013). There are important earlier models of homeostatic structural plasticity, such as the compensation model by Dammasch et al. (Dammasch et al., 1986, 1988; Cromme and Dammasch, 1989; Butz and Teuchert-Noodt, 2006; Butz et al., 2006) and the activity-dependent neurite outgrowth model by van Ooyen (van Ooyen and van Pelt, 1994; Van Ooyen et al., 1995). The latter model, which studied the reciprocal interactions between neuronal activity and network formation, successfully accounted for experimental data on developing cell cultures (van Ooyen and van Pelt, 1994; van Oss and van Ooyen, 1997; Abbott and Rohrkemper, 2007; Tetzlaff et al., 2010). However, both models are not suited for studying topology development. The compensation model lacks topology at all, whereas the representation of neuritic fields by circles in the model by van Ooyen imposes too strong constraints on network topology. In van Ooyen's model, neurons always connect to their direct neighbors before connecting to more distant neurons. Therefore, we developed our MSP (Butz and van Ooyen, 2013), in which we replaced the circle representation by discrete synaptic elements whose numbers change in an activity-dependent manner. Synapses are formed in a random and distance-dependent way by combining synaptic elements from different neurons.

In MSP, the local activity-dependent growth process in combination with a simple kernel function favoring the formation of short-range connections over long-range connections shapes the development of small-world networks. Our simulation results revealed an interesting property of homeostatic growth: as soon as most neurons approached homeostasis in electrical activity, they started forming more long-range connections than expected from the kernel function. Although the clustering coefficient decreased as a result of the formation of long-range connections, the network maintained its small-world property. Furthermore, connectivity became more diverse, as indicated by a decreasing betweenness centrality, and attained a higher global efficiency (defined as the averaged inverse of the shortest paths between all neurons in the network) than small-world networks without homeostasis. Our findings may account for experimental data on the topology of developing dissociated cell cultures (Downes et al., 2012). Interesting similarities were also found between the model and early human brain development (Gao et al., 2011) with respect to an increasing number of long-range connections and an increasing global efficiency during development.

## 2. Materials and methods

### 2.1. Neuron model

The model network consisted of *n* = 400 neurons, of which 80% were excitatory and 20% inhibitory. Excitatory neurons were placed with some jitter on a 20 × 16 grid with a spatial distance between two grid points of 150 μm. Inhibitory neurons were placed evenly between the excitatory neurons. The same network layout was used as in Butz and van Ooyen (2013). We used Izhikevich's model (Izhikevich, 2003) to simulate neuronal electrical activity. This model has two differential equations, one for the membrane potential *v* (in mV) and one for a recovery variable *u* (in mVms^−1^), enabling re-polarization after an action potential:

(1) $$\begin{array}{l} \frac{dv}{dt}=k_{1}v^{2}+k_{2}v+k_{3}-u+I\\ \frac{du}{dt}=a(bv-u) \end{array}$$

where *k*_1_ = 0.04 mV^−1^ ms^−1^, *k*_2_ = 5 ms^−1^, *k*_3_ = 140 mVms^−1^, and *t* is in ms. Every time a neuron fires (*v* ≥ 30 mV), *v* and *u* are reset:

(2) $$\text{if }v\geq 30\;\text{mV},\text{then }\{\begin{array}{ccc} v & \leftarrow & c\\ u & \leftarrow & u+d \end{array}$$

As in Izhikevich (2003), the following parameter values were used: *a* = 0.1 ms^−1^, *b* = 0.2 ms^−1^, *c* = −65 mV, *d* = 2 mVms^−1^. We used the same dynamics for excitatory and inhibitory neurons. Each neuron receives input *I* = *I_syn_* + *I_ext_*, consisting of synaptic input *I*_*syn*_ from within the network and external input *I*_*ext*_. Neurons interchange electrical signals on a millisecond timescale without synaptic delay. Synaptic input consists of the incoming action potentials from the presynaptic neurons low-pass filtered by an exponential filter function $h(t)=exp\left(-\frac{t}{\mu }\right)$ with decay constant μ = 5 ms. Network connectivity *W*_*i,j*_ is defined as the number of synapses from neuron *j* to *i*. If a synapse exists, it has a fixed strength of 1 mVms^−1^. Neurons are either excitatory or inhibitory. Indices refer to excitatory neurons if *i* or *j* ∈ {*Ex*} and to inhibitory neurons if *i* or *j* ∈ {*In*}. As in Izhikevich (2003) and Butz and van Ooyen (2013), *I*_*ext*_ is delivered as white noise with mean 5 mVms^−1^ and standard deviation 1 mVms^−1^. In some cases lower input values were required and specified where relevant.

The intracellular calcium concentration of a neuron is used as a low-passed filtered average of its firing frequency (Butz and van Ooyen, 2013). Every time a neuron fires, the calcium concentration is increased by a fixed amount; otherwise the concentration decreases exponentially to zero:

(3) $$\frac{d{\left[Ca^{2+}\right]}_{i}}{dt}=\;\{\begin{array}{cc} -\frac{{\left[Ca^{2+}\right]}_{i}}{\tau_{Ca}}+\beta & \text{if }v\geq 30\text{mV}\\ -\frac{{\left[Ca^{2+}\right]}_{i}}{\tau_{Ca}} & \text{otherwise} \end{array}$$

where β = 0.001 ms^−1^ and τ_*Ca*_ = 10,000 ms. We defined a set-point ϵ = 0.7 in calcium concentration, corresponding to an intermediate level of average electrical activity. Every time the neuron's calcium concentration deviates from ϵ, it will induce structural changes in connectivity to restore the desired average level of activity, as described below.

### 2.2. Synapse model for structural plasticity

During development, neurons show a pronounced formation and pruning of synapses. To simulate reorganization of synaptic connectivity, standard models of synaptic plasticity, in which connectivity is considered fixed with plasticity merely arising from changes in the strength of existing synapses (modeled as weight factors), are not suitable. For this study on the development of neuronal networks, we therefore used our Model of Structural Plasticity (MSP) (Butz and van Ooyen, 2013). The characteristic feature of this model is that it represents synapses as consisting of two recombinable synaptic elements, namely an axonal element and a dendritic element. Axonal elements represent countable presynaptic specializations for transmitter release such as axonal terminals or boutons, while dendritic elements represent the postsynaptic counterparts, i.e., postsynaptic receptor plates on dendritic spines or the dendritic shaft. Axonal and dendritic elements are either excitatory or inhibitory. Any neuron *i* can form *A_i_* axonal elements, which are excitatory if the neuron is excitatory, or inhibitory if the neuron is inhibitory. At the same time, a neuron, irrespective of its type, can express *D^ex^_i_* excitatory and *D^in^_i_* inhibitory dendritic elements. Complementary elements can merge to form a synapse (excitatory axonal with excitatory dendritic elements, and inhibitory axonal with inhibitory dendritic elements). Synaptic elements form and delete independently from a synaptic contact partner. In case a neuron deletes a synaptic element that is bound in a synapse, the complementary synaptic element on the other neuron remains and becomes vacant and available again for synapse formation with a new target. Therefore MSP allows for synaptic rewiring.

### 2.3. Homeostatic growth rules

**Figure 1:**
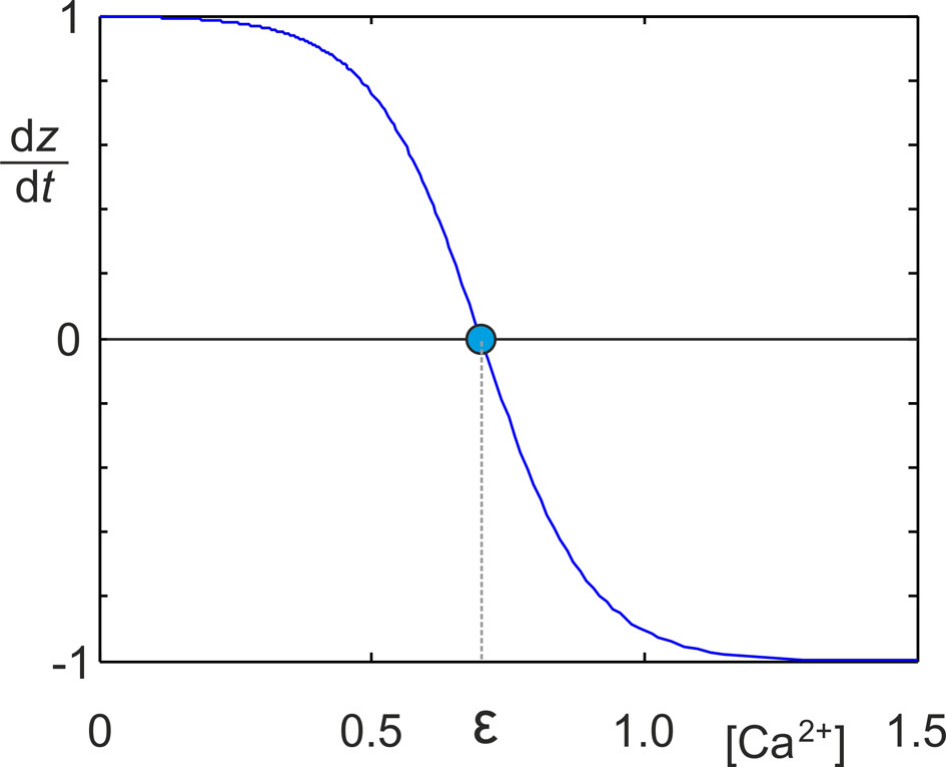
**Identical sigmoidal growth rules were used for all types of synaptic elements to determine the change *dz*/*dt* in the number of synaptic elements in dependence on the intracellular calcium concentration [*Ca*^2+^]**. *z* needs to be replaced by the respective type of element *A*, *D*^*ex*^ or *D*^*in*^. The homeostatic set-point ϵ is the value of the calcium concentration where *dz*/*dt* = 0.

It is well documented that neurons change their morphology in an activity-dependent fashion during development (Butz et al., 2009b; van Ooyen, 2011). The way in which neurons change their morphology suggests that they try to maintain homeostasis of electrical activity (van Ooyen and van Pelt, 1994; Van Ooyen et al., 1995; Butz et al., 2009a; van Ooyen, 2011; Butz and van Ooyen, 2013). That is, neurons in which the average activity is too low start forming new neuritic structures, whereas neurite growth is halted or neurites are pruned when activity is higher than a desired level (homeostatic set-point). In our MSP, we abstracted away from describing detailed neuronal morphology and assumed that changes in morphology effectively result in changing numbers of axonal and dendritic elements—the contact sites for synaptic connections. Homeostatic adaptation of the number of axonal and dendritic elements was modeled by the following growth rule (Figure 1), in which *dz* represents the change in the number of synaptic elements, with *z* being *A*, *D*^*ex*^ or *D*^*in*^:

(4) $$\frac{dz}{dt}=\nu \;\left(\frac{2}{1+e^{\left(\left[Ca^{2+}\right]-\epsilon \right)/0.1}}-1\right)$$

where ϵ is the set-point of the intracellular calcium concentration, corresponding to a desired average firing rate of the neuron, and ν is the growth rate of synaptic elements. We chose ν = 10^−4^ ms^−1^, which is slow enough that electrical dynamics and structural dynamics do not interfere, yet fast enough not to slow down the simulations unnecessarily. For reasons of simplicity and because of a lack of detailed experimental data, we applied identical sigmoid growth rules to all types of synaptic elements. In a random and distance-dependent recombination process, newly formed synaptic elements were distributed to matching synaptic elements, thereby forming synapses and creating the pattern of connectivity in the network.

### 2.4. Kernel function for synapse formation

As in our previous work on MSP (Butz and van Ooyen, 2013), we assumed that synapse formation is more likely between adjacent neurons than between distant ones. We applied a two-dimensional Gaussian-shaped kernel function centered at the *x*, *y*-coordinates of neuron *i*, with *K*_*i,j*_ the distance-dependent likelihood for synapse formation between neuron *j* and *i*:

(5) $$K_{i,j,\;i\;\neq \;j}=e^{-\frac{{\left(pos_{xj}-pos_{xi}\right)}^{2}+{\left(pos_{yj}-pos_{yi}\right)}^{2}}{\sigma^{2}}}$$

where *pos*_*xi*_ is the x-coordinate and *pos*_*yi*_ is the y-coordinate of postsynaptic neuron *i*, and *pos*_*xj*_ and *pos*_*yj*_ are the coordinates of presynaptic neuron *j*. The probability for autapse connections (i.e., a neuron connecting to itself) was set to zero (*K*_*i,j*_ = 0 for *i* = *j*). For these simulations we chose σ = 1 × 150 μm where 150 μm is the distance between two grid points.

In order to investigate the impact of this distance-dependency on emerging network topologies, we additionally grew networks with a flat kernel, i.e., with *K*_*i,j, i* ≠ *j*_ = 1 and *K*_*i,j*_ = 0 for *i* = *j*.

### 2.5. Synapse formation and deletion

The MSP algorithm (Butz and van Ooyen, 2013) proceeded in three steps to update network connectivity in an activity-dependent fashion. First, electrical activity of all neurons was computed continuously over time. Secondly, depending on the average electrical activity and according to the homeostatic growth rule (Equation 4), the number of elements changed continuously, too. Thirdly, at discrete time points, network connectivity *W* was updated by synapse formation and deletion. Because of the low growth rate ν = 10^−4^ ms^−1^, changes in connectivity are very slow compared to changes in activity, so it was not necessary to update connectivity at every time step but only at every 100 ms. The timescale of network formation in the model corresponds in principle to a timescale of days or weeks. However, as described above under homeostatic growth rules, the timescale of structural changes was chosen so that the simulations were not unnecessarily slowed down, which means that the total duration of the simulation in milliseconds is not required to sum up to weeks.

#### 2.5.1. Synapse deletion

Since network connectivity is updated at discrete time steps but synaptic elements change continuously over time due to the activity-dependent growth rules, it can happen that a neuron has more outgoing synapses than axonal elements or more incoming synapses than dendritic elements at the time of the next update in network connectivity. In this case, the neuron has to delete the surplus of synapses and to update connectivity.

To update connectivity, the algorithm needs to select which synapses are to be removed. All synapses have an equal chance of being deleted. Note, however, that multiple synapses can co-exist from neuron *j* to *i* and that the more synapses there are, the higher the chance that a synapse between neuron *j* and *i* will be deleted. The probability *P*^*del*^_*i,j*_ for synapse deletion between neuron *j* and *i* is computed by the following master equation that captures four different cases:

(6) $$P_{i,j}^{del}=\frac{W_{i,j}}{\sum W_{k,l}}$$

For deletion of incoming synapses, we need to distinguish between excitatory and inhibitory synapses in Equation (6). For deleting incoming excitatory synapses of neuron *i* ∈ {*In* ∪ *Ex*}, we sum up *W*_*k,l*_ over all *l* ∈ {*Ex*}. For deleting incoming inhibitory synapses of neuron *i* ∈ {*In* ∪ *Ex*}, we sum up *W*_*k,l*_ over all *l* ∈ {*In*}. For deletion of outgoing excitatory synapses of excitatory presynaptic neuron *j* with *j* ∈ {*Ex*}, in Equation (6) all synapses are considered to any postsynaptic neuron *k* with *k* ∈ {*In* ∪ *Ex*}. Thus, we sum up *W*_*k,l*_ over all *k* ∈ {*In* ∪ *Ex*}. The same holds true for outgoing inhibitory synapses with *j* ∈ {*In*}.

Sequentially, outgoing and incoming excitatory and inhibitory synapses were selected for deletion. For every type of synapse, the accumulated sum of *P*^*del*^_*i,j*_ [see description of Equation (6) for the range of *i* and *j*] gave a probability distribution from which we drew the required number of synapses to be deleted. The selected synapse was deleted by reducing the respective entry *W*_*i,j*_ in the connectivity matrix by one. It can happen that more then one synapse is selected for deletion from the same connection *j* to *i*. In this case, the implementation of the algorithm makes sure that the number of synapses to be deleted did not exceed *W*_*i,j*_. Whenever a neuron deletes a synaptic element that is bound in a synapse, the complementary synaptic element on the other neuron remains and becomes vacant again.

#### 2.5.2. Synapse formation

For synapse formation, the algorithm checked whether a neuron gained vacant synaptic elements, i.e., whether the total number of synaptic elements exceeded the number of bound synaptic elements of this type. Matching vacant synaptic elements (vacant excitatory axonal elements *A*^*ex,vac*^_*j*_ with vacant excitatory dendritic elements *D*^*ex,vac*^_*i*_, and inhibitory axonal with inhibitory dendritic elements *D*^*in,vac*^) were randomly distributed among each other with probability density function *P*^*form*^. The probability *P*^*form*^_*i,j*_ for forming new synapses between neuron *j* and *i* depended on the number of vacant synaptic elements they offered and on the Euclidean distance between neuron *j* and *i*:

(7) $$P_{i,j}^{form}=\left\{\begin{array}{cc} j\in \{Ex\}\;:\; & \frac{A_{j}^{vac}\;D_{i}^{ex,vac}}{\sum_{\iota \in \{Ex\}}A_{\iota }^{vac}\;\sum_{\kappa \in \{Ex\cup In\}}D_{\kappa }^{ex,vac}}\;K_{ij}\\ j\in \{In\}\;:\; & \frac{A_{j}^{vac}\;D_{i}^{in,vac}}{\sum_{\iota \in \{In\}}A_{\iota }^{vac}\;\sum_{\kappa \in \{Ex\cup In\}}D_{\kappa }^{in,vac}}\;K_{ij} \end{array}\right\}\text{with }i\in \{Ex\cup In\}.$$

The minor number of vacant excitatory and inhibitory axonal or dendritic elements determined how many new excitatory and inhibitory synapses, respectively, could at most be formed (so-called potential synapses) in every update of connectivity. Thus, the number of excitatory and inhibitory potential synapses equaled

(8) $$\begin{array}{c} M^{PotSyn,ex}=min\left(\sum_{\iota \in \{Ex\}}A_{\iota }^{vac},\;\sum_{\kappa \in \{Ex\cup In\}}D_{\kappa }^{ex,vac}\right)\\ M^{PotSyn,in}=min\left(\sum_{\iota \in \{In\}},\;A_{\iota }^{vac},\;\sum_{\kappa \in \{Ex\cup In\}}D_{\kappa }^{in,vac}\right) \end{array}$$

for every update in connectivity.

From this distribution, the algorithm chose at maximum *M*^*PotSyn,ex*^ excitatory and *M*^*PotSyn,in*^ inhibitory connections at which new synapses were created. The respective entries *W*_*i,j*_ in the connectivity matrix were then increased by one. A connection was chosen by drawing a random number from a uniform distribution and comparing it to the accumulated probabilities *P*^*form*^_*i,j*_ for all excitatory connections and all inhibitory connections of the entire network. That connection was chosen that had the highest accumulated probability that the random number just did not exceed. If, for this try, the random number exceeded all accumulated probabilities, no synapse was formed. Hence, not necessarily all of the potential synapses were formed.

Additionally, synapse formation needed to fulfil the condition that the number *W*^+^_*i,j*_ of newly-formed synapses from neuron *j* to *i* did not exceed the number of vacant synaptic elements that neuron *j* and *i* offered:

(9) $$W_{i,j}^{+}\leq \left\{\begin{array}{lll} j\in \{Ex\} & : & \min(A_{j}^{vac}\;,D_{i}^{ex,vac})\\ j\in \{In\} & : & \min(A_{j}^{vac}\;,D_{i}^{in,vac}) \end{array}\right\}\text{with }i\in \{Ex\cup In\}.$$

In every update, this condition was checked and synapse formation infringing this condition was rejected. Alternatively, the update of connectivity can also be implemented in a purely local fashion (Butz and van Ooyen, 2013). For small networks, the current implementation is more efficient than the original MSP algorithm (Butz and van Ooyen, 2013) because run time is not dependent on the growing numbers of vacant synaptic elements but proportional to the square of the number of neurons in the network. However, for large networks with *n* >> 1000, *n*^2^ will quickly out-range the number of vacant synaptic elements, in which case looping over the number of synaptic elements would be faster. Particularly in Matlab, the current description of MSP allows for an elegant vectorized implementation providing additional speed up.

### 2.6. Development of non-homeostatic networks

To investigate the impact of homeostasis in electrical activity on developing network topology, we created for every homeostatic network a corresponding non-homeostatic network. At every update in connectivity, we took the number of synapses from the homeostatic network and distributed them in the non-homeostatic network with the same kernel function as used in the homeostatic network. Hence, the placement of synapses in the non-homeostatic network was purely dependent on the kernel function but did not meet the homeostasis criterion. The algorithmic implementation for placing synapses was the same for homeostatic and non-homeostatic networks, with *P*^*form*^_*i,j*_ = *K*_*i,j*_. Instead of distributing *M*^*PotSyn*^ synapses (Equation 8), we simply distributed the total number of synapses from the homeostatic network. Since synapse formation was not limited by numbers of vacant elements, Equation (9) was not applicable.

### 2.7. Topology measurements

A neuronal network can be seen as a graph with the neurons being the nodes and the synapses being the edges or links between two nodes. Since the presynaptic neuron always activates the postsynaptic neuron (and never the other way around), we regard the graph as directed. At every update in connectivity, we assessed those graph theoretical measures that are indicative of small-worldness and network efficiency. In addition, betweenness centrality was measured to determine the importance of nodes in the network. To reduce the complexity of the assessment, we considered only the topology of excitatory synaptic connections *W*^*ex,ex*^ between the *n*^*ex*^ excitatory neurons. For the graph theoretical assessments, the brain connectivity toolbox by Olaf Sporns et al. (Rubinov and Sporns, 2010) was used.

#### 2.7.1. Weighted characteristic path length

The characteristic path length *L* is the average shortest path from one node to any other node in the network. Path length is defined as the number of links that need to be traveled to go from one node (possibly via intermediate nodes) to any other node. On top of this definition, a direct link between two nodes in a weighted network is considered “shorter” the stronger the weight of the link is. For our network, we take the number of synapses *W*^*ex,ex*^_*i,j*_ between two directly linked neurons *j* and *i*, with *i, j* ∈ {*Ex*}, as the weight of the connection and the inverse 1/*W*^*ex,ex*^_*i,j*_ as the length *l*_*i,j*_ of the connection. The shortest path *d*_*i,j*_ is then the smallest sum of connection lengths that lead from neuron *j* to *i* via any intermediate neurons. Our calculation of the weighted characteristic path length was based on an implementation of Dijkstra's algorithm for computing the shortest path in weighted directed networks by Rubinov and Sporns (2010).

#### 2.7.2. Weighted clustering coefficient

The clustering coefficient is an indication of how strongly nodes in a network are interconnected. It can be measured by the number of triangles, ${\tilde{t}}_{i}^{D}$, one node forms with any other two nodes in the network divided by the total number of possible triangles, *T^D^_i_*. For weighted and directed graphs, one needs to realize that the adjacency matrix (in our case, the connectivity matrix *W*^*ex,ex*^ of the excitatory neurons) is not symmetric and that the entries of the adjacency matrix are not one but can have any weight. According to Fagiolo (2007), the clustering coefficient of a single node in a weighted directed network ${\tilde{C}}_{i}^{D}$ is computed as

(10) $${\tilde{C}}_{i}^{D}\left(W^{ex,ex}\right)=\frac{{\tilde{t}}_{i}^{D}}{T_{i}^{D}}=\frac{{\left[{\left(W^{ex,ex}\right)}^{\left[1/3\right]}+{\left({\left(W^{ex,ex}\right)}^{T}\right)}^{\left[1/3\right]}\right]}_{i,i}^{3}}{2\left[d_{i}^{tot}\left(d_{i}^{tot}-1\right)-2d_{i}^{↔}\right]}$$

where (*W^ex,ex^*)^[1/*k*]^ = *w*^1/*k*^_*i,j*_, the *k*^*th*^ root of the entries of the matrix for *i,j* ∈ {*Ex*}, and (*W^ex,ex^*)^*T*^ is the transposed *W*^*ex,ex*^ matrix. The variable *d*^*tot*^_*i*_ denotes the total degree of node *i* (the degree counts the number of either incoming or outgoing edges per node, and the total degree is the sum over both the incoming and outgoing edges), and *d*^↔^_*i*_ stands for the number of bilateral edges of node *i* (the number of nodes node *i* projects to and receives edges from, excluding self-interactions of node *i*).

The overall clustering coefficient of the network is thus ${\tilde{C}}^{D}={\left(n^{ex}\right)}^{-1}\sum_{i\;=\;1}^{N}{\tilde{C}}_{i}^{D}$. Note that for this assessment we only considered *n*^*ex*^ excitatory nodes and excitatory connections *W*^*ex,ex*^. For a more detailed description of Equation (10), see Fagiolo (2007). We computed the clustering coefficient of the developing neuronal networks at every update in connectivity by using the implementation by Mika Rubinov from the brain connectivity toolbox (Rubinov and Sporns, 2010).

#### 2.7.3. Small-world parameter

To estimate small-worldness of the developing networks, we applied the formalism by Humphries and Gurney (2008):

(11) $$s=\frac{C/C^{rand}}{L/L^{rand}}$$

We replaced the clustering coefficient *C* and the characteristic path length *L* by the corresponding versions for weighted directed graphs as described above. *C*^*rand*^ and *L*^*rand*^ were taken from an Erdős-Rényi random graph generated with the same number of synapses as in the developing networks at every update in connectivity.

#### 2.7.4. Betweenness centrality

The local betweenness centrality is a measure for the importance of a node in a network. A high betweenness centrality of a node *i* means that many shortest paths between any two nodes *k* and *l* pass through node *i*. Thus, the local betweenness centrality counts the number of times, σ_*kl*_(*i*), that node *i* is on a shortest path between two nodes *k* and *l*. The local betweenness centrality is normalized by the number σ_*kl*_ of alternative shortest paths between *k* and *l* that do not pass through node *i*. Global betweenness centrality is the sum of all local betweenness centrality values of the individual nodes:

(12) $$BC_{global}=\sum_{i}^{n^{ex}}\sum_{k\;\neq \;i\;\neq \;l}\frac{\sigma_{kl}(i)}{\sigma_{kl}}$$

Consequently, betweenness centrality provides a measure for how well networks are interconnected. A high local or global betweenness centrality means that individual nodes or the entire network, respectively, is badly interconnected, because all information has to travel through the same nodes in the absence of alternative routes or by-passes. Clinical data shows that after brain lesions, betweenness centrality of directly and indirectly affected brain areas changes (Wang et al., 2010). Note that as for the measurements above, the shortest paths are based on weighted excitatory connections *W*^*ex,ex*^_*i,j*_. Therefore, global betweenness centrality was computed by the formalism for weighted directed networks by Brandes (2001) as implemented in the brain connectivity toolbox (Rubinov and Sporns, 2010).

#### 2.7.5. Efficiency

Global efficiency is related to the inverse characteristic path length with the advantage over characteristic path length that it can be meaningfully computed also of disconnected graphs (Latora and Marchiori, 2001; Achard and Bullmore, 2007). While path lengths between disconnected cells are infinite, efficiency becomes zero and, thus, adds neutrally to global efficiency.

(13) $$E_{global}=\frac{1}{n^{ex}\left(n^{ex}-1\right)}\sum_{i\;\neq \;j}\frac{1}{d_{i,j}}$$

where *n*^*ex*^ is the number of excitatory neurons.

#### 2.7.6. Euclidean distance

Although not a topology measure in a strict graph theoretical sense, the average Euclidean distance between nodes that are connected by synapses was measured in order to help interpret the development of characteristic path length and clustering coefficient. To obtain the average Euclidean distance *ED*, we multiplied the Euclidean distances between all pairs of excitatory neurons in the network with the number of synapses between these neurons. We then summed up all of the so-weighted distances and divided the sum by the number of excitatoy synapses:

(14) $$ED=\left(\sum_{i,j}^{n^{ex}}\sqrt{{\left(pos_{xj}-pos_{xi}\right)}^{2}+{\left(pos_{yj}-pos_{yi}\right)}^{2}}W_{i,j}^{ex,ex}\right)/\sum_{i,j}^{n^{ex}}W_{i,j}^{ex,ex}$$

with *i,j* ∈ {*Ex*}

## 3. Results

**Figure 2:**
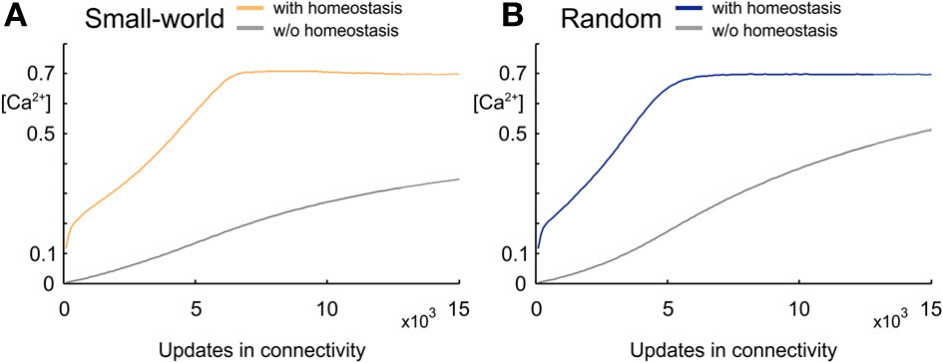
**Development of intracellular calcium concentration [*Ca*^2+^] over time. (A)** Development of calcium concentration in small-world networks arising from synapse formation that favors short-range over long-range connections. Mean calcium concentrations averaged over five simulations reach the set-point ϵ = 0.7 when the number of synaptic elements is regulated homeostatically (yellow), whereas calcium concentrations remain much lower when there is no homeostatic regulation (gray). **(B)** Development of calcium concentration in random networks without any distance-dependency in synapse formation. The random network with homeostatic regulation of synaptic elements (blue) also reaches the homeostatic set-point ϵ, whereas the network without homeostasis (gray) and the same number of synapses as the homeostatic network has much lower values in calcium. Shadow around the curves (hardly visible since so small) indicates the standard deviation.

**Figure 3:**
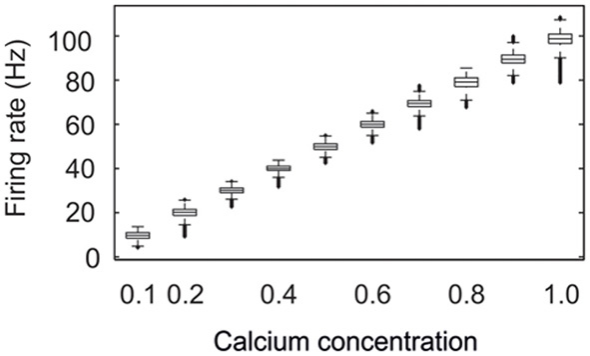
**Neurons develop their connectivity in order to reach a homeostatic set-point ϵ of intracellular calcium**. The figure shows the firing rates attained for different set-point values of calcium. For each calcium concentration, the firing rates of all neurons were pooled from four different simulations recorded over the last 20,000 ms. The central mark of each box is the median firing rate; boxes represent the 25th and 75th percentiles; the whiskers extend to the most extreme data points not considered “outliers”; and “outliers” are plotted individually (which show up here as thick bold lines). An “outlier” is a value that is more than 1.5 times the interquartile range away from the top or bottom of the box. The firing rate is proportional to ϵ by a factor of 100. For this set of simulations lower external inputs *I*^*ext*^ with mean 2 mVms^−1^ were used.

We started each network simulation with zero connectivity and zero synaptic elements. Due to the homeostatic formation of axonal and dendritic elements, model neurons are able to form synapses and to adapt the number of synapses so as to reach a homeostatic equilibrium in electrical activity (Figure 2). For a wide range of set-points ϵ, model neurons adapt their average firing rate so that, at the end of each simulation (here after 15,000 updates in connectivity), their calcium concentration [*Ca*^2+^] has converged to ϵ, with a corresponding firing rate *y* (in Hz) that follows the linear relation *y* = 100 × [*Ca*^2+^] (Figure 3). We investigated how the topology of these self-organizing networks developed over time when neurons favor short-range connections over long-range connections, or, alternatively, when all connections are equally likely. In the first case, as we will show in detail later on, networks developed a distinct small-world property, with small-world parameter *s* markedly greater than 1, whereas in the second case, *s* reaches 1. Therefore, we will call networks that resulted from distance-dependent synapse formation small-world networks and those that resulted from synapse formation without distance-dependency random networks.

**Figure 4:**
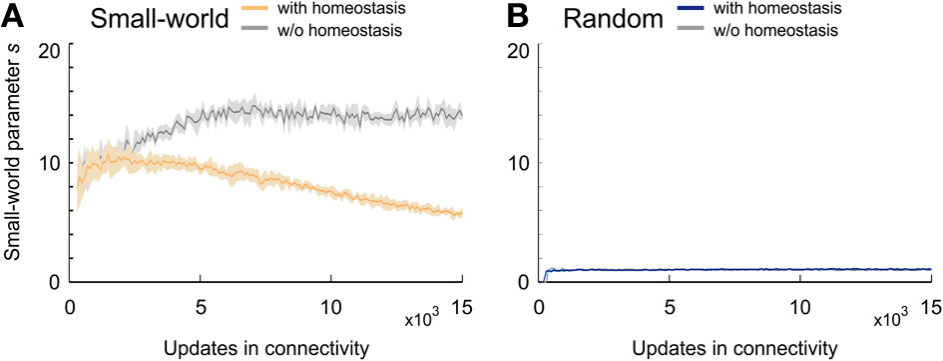
**Small-worldness of developing networks. (A)** Small-world networks with (orange) and without (gray) homeostasis in electrical activity. **(B)** Random networks with (blue) and without (gray) homeostasis in electrical activity. Means over five simulations per scenario. Shadings of the curves indicate standard deviations.

In networks that favor long-range over short-range connections (small-world networks) (Figure 4A), *s* constantly increased and reached a maximum markedly greater than 10 at around 7000 updates in connectivity. Small-world networks that were set-up by an additional rule for homeostasis in electrical activity reached a plateau of about *s* = 10 very early but began to decrease again around the time that neuronal activities reached the homeostatic set-point ϵ. Nevertheless, small-world networks with homeostasis maintained their small-world property throughout the whole course of the simulation (*s* > 5 at *T* = 15,000). In networks without distance-dependency in synapse formation (random networks) (Figure 4B), *s* equaled 1 from the very beginning of network development. Random networks with and without homeostasis did not differ in the course of *s* (Figure 4B). Hence, homeostasis did not seem to have an impact on the development of topology in random networks. Knowing that homeostasis influenced the development of small-worldness, we further analyzed how homeostasis exerted its influence during network formation. For some simulations, *s* could not be computed for the first few updates in connectivity because of division by zero.

### 3.1. Homeostasis influences the clustering coefficient and characteristic path length of developing small-world networks

**Figure 5:**
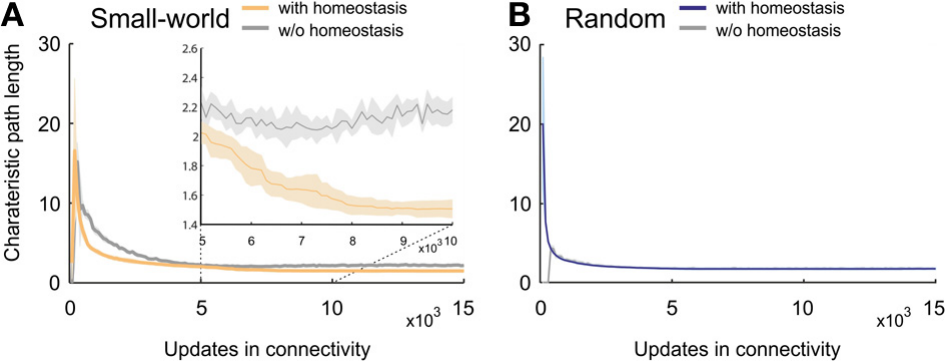
**Characteristic path length in developing networks. (A)** Small-world networks with (orange) and without (gray) homeostasis in electrical activity. The inset in **(A)** is a close-up of the time interval from 5000 to 10,000 updates in connectivity that clearly shows the decay in characteristic path length in homeostatic networks compared to non-homeostatic networks. **(B)** Random networks with (blue) and without (gray) homeostasis in electrical activity. Means over five simulations per scenario. Shadings of the curves indicate standard deviations.

The mean or characteristic path length in small-world networks without homeostasis showed, after an initial sharp rise, a steady decrease and converged toward values of around 3 (Figure 5A). Homeostatic small-world networks also started with a sharp rise and a subsequent decrease in characteristic path length. The decrease was even steeper than in the non-homeostatic case, and the characteristic path length converged toward slightly lower values than in non-homeostatic networks. In addition, networks with homeostasis showed a second but minor decrease in characteristic path length when activities reached the homeostatic set-point ϵ. The final values of the characteristic path length after 15,000 updates in connectivity were stable in both homeostatic and non-homeostatic small-world networks. The initial sharp increase is caused by the limited number of synapses in the network at the beginning of the simulation. Characteristic path lengths in developing random networks showed an identical course with and without homeostasis (Figure 5B). The final values in random networks were marginally lower than those in homeostatic small-world networks.

**Figure 6:**
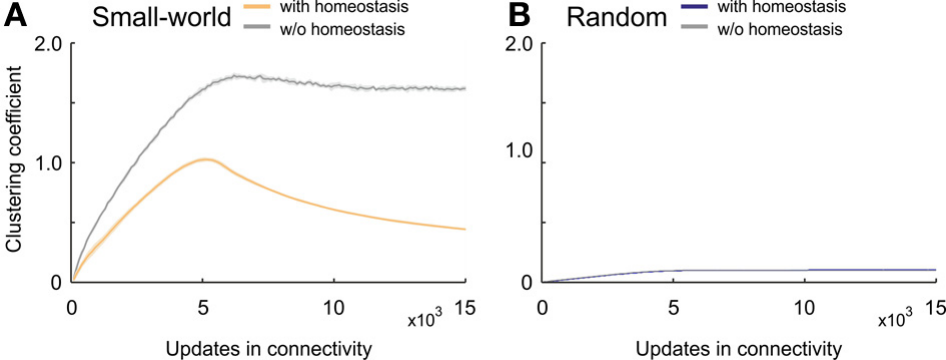
**Clustering coefficient in developing networks. (A)** Small-world networks with (orange) and without (gray) homeostasis in electrical activity. **(B)** Random networks with (blue) and without (gray) homeostasis in electrical activity. Means over five simulations per scenario. Shadings of the curves indicate standard deviations.

The clustering coefficient in developing homeostatic small-world networks took a considerably different course from the coefficient in small-world networks without homeostasis (Figure 6A). Whereas the clustering coefficient in networks without homeostasis converged, with a small overshoot, toward high levels of over 1.6, networks with homeostasis generated a considerable overshoot and ended up at much lower values as compared with non-homeostatic networks. After a ramp-up phase, homeostatic networks reached a maximum clustering coefficient of about one; thereafter, the clustering coefficient decreased again with a decreasing negative slope. The maximum clustering coefficient was reached when the average electrical activities approached the homeostatic set-point ϵ. By contrast, we did not see an overshoot in clustering coefficient in homeostatic and non-homeostatic random networks (Figure 6B). Therefore, the homeostasis in electrical activity had no effect on clustering in random networks.

### 3.2. Homeostasis favors long-range connections in small-world networks

**Figure 7:**
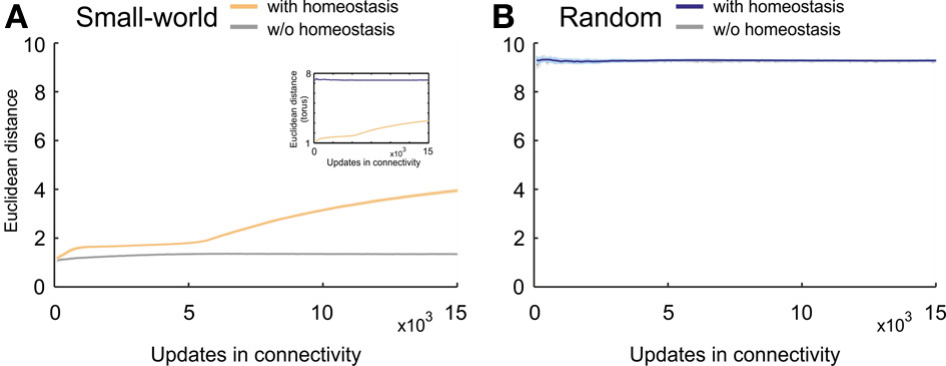
**Average Euclidean distance between pre- and postsynaptic neurons for every synapse during network development. (A)** Small-world networks with (orange) and without (gray) homeostasis in electrical activity. **(B)** Random networks with (blue) and without (gray) homeostasis in electrical activity. Means over five simulations per scenario. Shadings of the curves indicate standard deviations. Inset in **(A)** depicts the change in Euclidean distances for homeostatic random (blue) and homeostatic small-world networks (orange) with torus boundary conditions.

Particularly the development of the clustering coefficient, with its pronounced overshoot, determines the emerging small-worldness in networks favoring short-range connections over long-range connections. We therefore further studied how homeostasis influenced clustering in small-world networks. We tested the hypothesis that homeostasis produced more long-range connections than expected from the kernel function *K* (Equation 5). Computing the average Euclidean distance between the pre- and postsynaptic neuron for every synapse indeed revealed longer average Euclidean distances in homeostatic than in non-homeostatic networks (Figure 7A). In non-homeostatic networks, the average Euclidean distance was constant, because it is directly derived from the kernel function (in Equation 5: σ = 150 μm). In homeostatic networks, however, we observed two different phases. First, during initial network development ([*Ca*^2+^] << ϵ), the average Euclidean distance converged quickly toward a stable value of around 2, which was only slightly higher than in non-homeostatic networks. Secondly, when calcium concentrations approached the homeostatic set-point ϵ, the average Euclidean distances ramped up and reached values greater than 4. The considerable increase in the average Euclidean distance of synaptic connections coincided with a drop in clustering coefficient. As the initial high clustering of the network is due to the kernel function for synapse formation favoring short- over long-range connections, we may conclude that increasing Euclidean lengths of synaptic connections give rise to the decrease in clustering right at the time neurons approach the homeostatic set-point in electrical activity. The effect was also noticeable in the course of the characteristic path length but much less pronounced. We did not observe a comparable effect of homeostasis on Euclidean distances of synaptic connections in random networks (Figure 7B).

**Figure 8:**
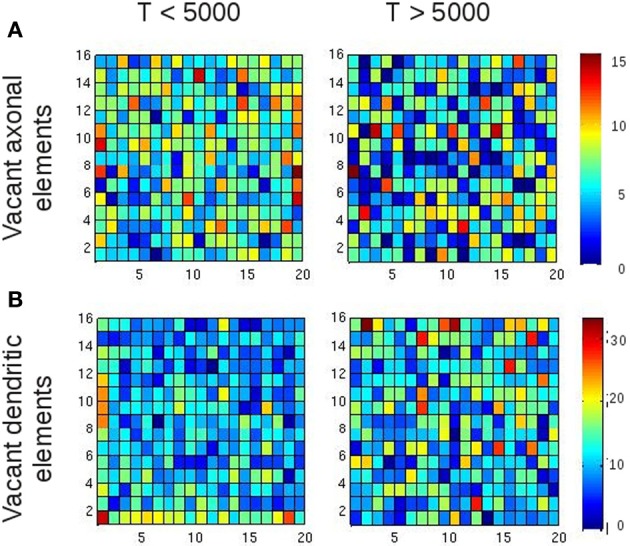
**Spatial position of vacant synaptic elements in small-world networks**. The purpose of this figure is to rule out that the position of vacant synaptic elements alone gave rise to longer-ranged synaptic connections. The left column shows vacant synaptic elements accumulated over the first 5000 updates in connectivity, whereas the right column shows the accumulation of vacant synaptic elements over the following 5000 updates. Although some tendency of vacant synaptic elements being located at the borders of the network is visible, this effect is gone after 5000 updates in connectivity. So there is no bias in the distribution of vacant synaptic elements when activity reaches the homeostatic set-point, and therefore the position of vacant synaptic elements alone cannot account for the formation of longer-ranged synaptic connections for *T* > 5000. **(A)** Vacant axonal elements. **(B)** Vacant dendritic elements. Color scale indicates number of vacant synaptic elements per neuron.

Are the increasing average Euclidean distances of synaptic connections caused by the fact that average neuronal activities are reaching a homeostatic equilibrium, or is this just some network effect that merely coincides with neurons equilibrating their activities? To answer this question, we assessed the number of all types of vacant elements (i.e., excitatory or inhibitory axonal elements, excitatory or inhibitory dendritic elements) and the spatial position of their hosting neurons at every time step of the simulation. We first checked whether there was any bias in the spatial position of synaptic elements that could generate more distant connections, e.g., a placement of synaptic elements at the boundaries of the network. We accumulated the number of vacant axonal (Figure 8A) and dendritic elements (Figure 8B) for each neuron for the first 5000 updates in connectivity with [*Ca*^2+^] < ϵ as well as for the next 5000 time steps with [*Ca*^2+^] ≈ ϵ. In the beginning of the simulation, we indeed found a little more vacant synaptic elements at the network boundaries, which can be explained by the fact that neurons at boundaries have less neighbors than neurons in the center and compensate for this by getting a higher number of vacant dendritic elements. However, by the time that the homeostatic equilibrium is reached, vacant synaptic elements were equally distributed over the network, and therefore the placement of vacant synaptic elements cannot account for the increasing distances of synaptic connections once the network has reached the homeostatic set-point. We found a comparable course of Euclidean distances in homeostatic small-world networks with torus boundary conditions (see inset of Figure 7A).

**Figure 9:**
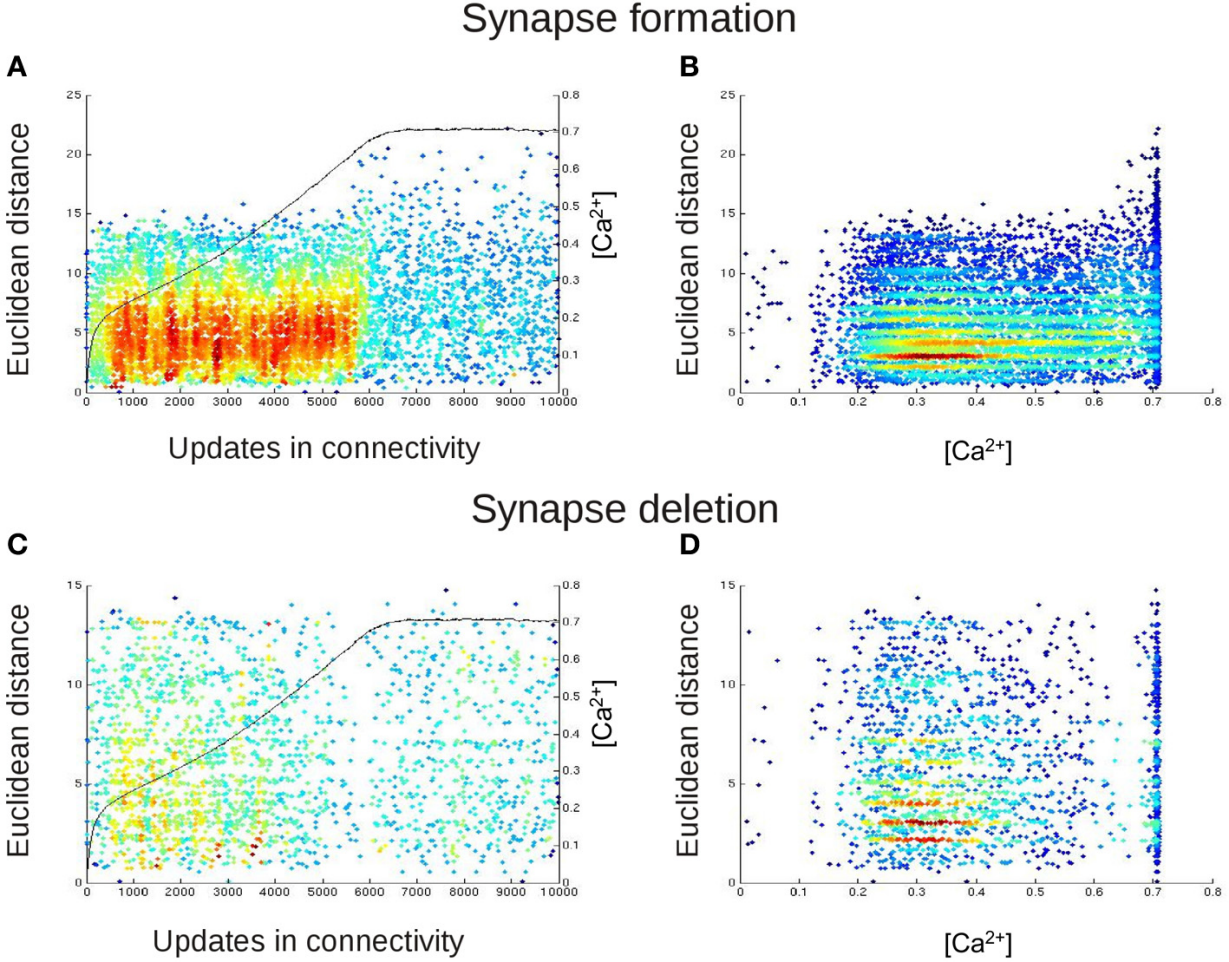
**The spatial distribution of newly formed synapses change in dependence on the calcium concentration**. In **(A,B)**, each dot represents the Euclidean distances between those neurons that are most likely to form a synaptic connection with each other at this update in connectivity. For this, we took at every update in connectivity in which vacant synaptic elements were available the Euclidean distance of the connection from neuron *j* to *i* for which *P*^*form*^_*i,j*_ (Equation 7) was maximal. In **(C,D)**, each dot represents the length of that connection (again in terms of the Euclidean distance between the connected neurons) for which synapse deletion was most likely, i.e., *P*^*del*^_*i,j*_ (Equation 6) was maximal for every update in connectivity in which synapses had to be deleted. In **(A,C)**, we plotted the Euclidean distances for synapse formation and deletion over time. The black curve (right y-axis) indicates the course of the calcium concentration [*Ca*^2+^]. In **(B,D)**, we plotted synapse formation and deletion in dependence on [*Ca*^2+^]. The color code in all panels indicates the density of the dots in the diagrams, with blue and red representing low and high densities of dots, respectively. The figure essentially shows that before calcium reaches the homeostatic set-point ϵ, the distribution for synapse formation is rather Gaussian, following the Kernel function *K* (Equation 5). The distribution becomes random and scattered, with increased Euclidean distances, when calcium is at the set-point. The stripes in the distribution arise from the fact that not all Euclidean distances are possible due to the grid layout of the network. There is no change in the distribution for synapse deletion.

Since we could exclude a spatial bias in the distribution of vacant synaptic elements, we tested whether the increasing distances of synaptic connections were a direct consequence of the activity-dependent growth rules. On the basis of the number of vacant elements per neuron and the Euclidean distance between the host neurons, we determined the most likely synaptic connection every time a new synapses was formed, which is equivalent to the maximum of *P*^*form*^_*i,j*_ (Equation 7). Since the change in the number of synaptic elements and therefore also the distribution of vacant synaptic elements are activity-dependent, the most likely synaptic connection to be formed is a direct consequence of the neurons' electrical activities. It turned out that in the beginning of development, when all neurons offer vacant synaptic elements, the most likely synaptic connections are those between adjacent neurons (Figures 9A,B). In other words, when neuronal activities were much lower than the homeostatic set-point ϵ, the kernel function had a large impact on the Euclidean length of synaptic connections. Therefore, at this stage of development, homeostatic and non-homeostatic networks did not differ much in connection lengths. However, when the activity of all individual neurons approached the set-point ϵ, vacant synaptic elements became rare and matching synaptic elements were available, if at all, only between more distant neurons. Expected distances for most likely synaptic connections therefore became much larger. Moreover, the distribution of expected Euclidean lengths of new synaptic connections did not follow the Gaussian-shaped kernel any longer but became much wider and flatter. At the same time, it took much longer for a synapse to form because, although longer-range synaptic connections were the most likely ones, their probability was still very low due to the kernel function. Due to a lack of shorter-range alternatives, these longer-range synaptic connections were nonetheless formed at some point in time, because the kernel function is non-zero for all distances. Taken together, the Euclidean length of synaptic connections in small-world networks is influenced by the homeostatic formation of synaptic elements. As expected from the topology data, no activity-dependent effect on the Euclidean distance of synaptic connections was observed in random networks.

Additionally, synapse deletion could in principle also influence the Euclidean length of synaptic connections if, for example, with increasing neuronal activities preferentially short connections would be deleted. Therefore, we further tested whether the expected Euclidean length of synaptic connections that were most likely to be deleted correlated with the current average activity in the network. However, an activity-dependent effect on synapse deletion was not observed (Figures 9C,D).

### 3.3. Homeostasis decreases the betweenness centrality in small-world networks

**Figure 10:**
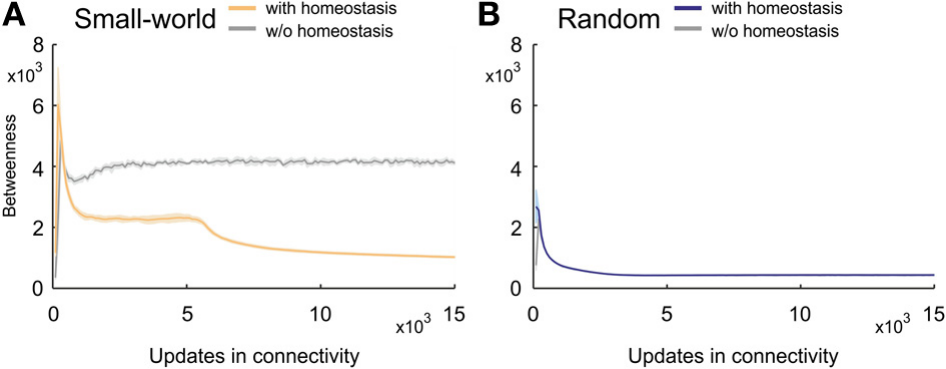
**Changing betweenness centrality over time. (A)** Small-world networks with (orange) and without (gray) homeostasis in electrical activity. **(B)** Random networks with (blue) and without (gray) homeostasis in electrical activity. Means over five simulations per scenario. Shadings of the curves indicate standard deviations.

Betweenness centrality (or inbetweenness) is a measure for the importance of nodes in a network. Compared with random networks, small-world networks without homeostatic synapse formation had a relatively high betweenness centrality (Figure 10A). Small-world networks with homeostasis, by contrast, revealed a pronounced decrease in betweenness centrality over developmental time. Values peaked in the beginning of development when neurons first connected to their nearest neighbors only. However, right after the peak, homeostatic synapse formation generated topologies with values for betweenness centrality that were lower than in non-homeostatic networks. Betweenness centrality is high in non-homeostatic small-world networks because synapse formation is only determined by the kernel function (Equation 5), which often caused that the same cell pairs were connected repeatedly. The consequence is a limited number of shortest paths between any pairs of nodes in the network. By contrast, in homeostatic networks, synapse formation depends on the availability of synaptic elements, which can force synapses to be formed that are less likely according to the kernel. This may increase the number of alternative paths between two neurons and therefore also increase the number of multiple shortest paths through the network. Hence, betweenness centrality quickly decreased over time.

Because the variety of shortest paths in non-homeostatic networks is limited, betweenness centrality reached a stable plateau in these networks. Interestingly, the betweenness centrality in homeostatic networks initially also converged toward a quasi-stable level. However, as soon as activities approached the homeostatic set-point ϵ, betweenness centrality strongly decreased. Over time, the rate of decrease slowed down. The decrease in betweenness centrality precisely coincided with the increase in Euclidean lengths of synaptic connections. Because the kernel function favors short- over long-range connections and therefore initially creates networks with high betweenness centrality, we may conclude that the formation of longer-range connections (in an Euclidean sense) lead to a decreasing betweenness centrality. Any new long-range connection not present in the network before creates new shortest paths, which in turn decreases betweenness centrality. By contrast, homeostasis did not affect the course of betweenness centrality in random networks (Figure 10B).

### 3.4. Small-world networks become more efficient by homeostasis

**Figure 11:**
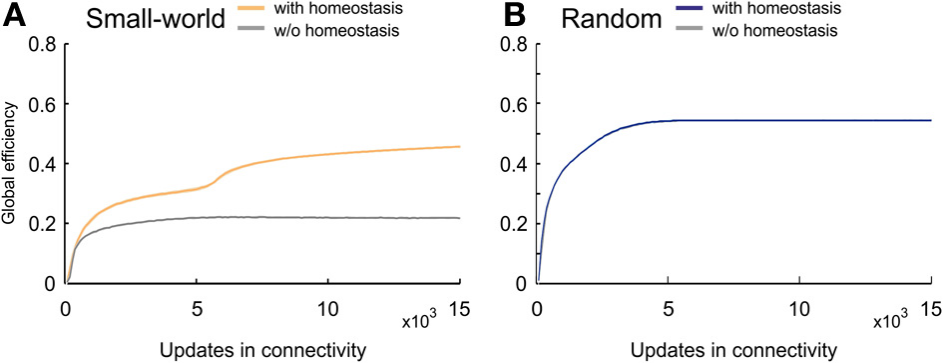
**Global efficiency changes over time due to network development. (A)** Small-world networks with (orange) and without (gray) homeostasis in electrical activity. **(B)** Random networks with (blue) and without (gray) homeostasis in electrical activity. Means over five simulations per scenario. Shadings of the curves indicate standard deviations.

Efficient information transmission is probably the most-desired property in computational networks. Small-world networks are very efficient because they combine a high clustering coefficient with a short characteristic path length. Nevertheless, their efficiency is still markedly lower than that of random networks. Homeostatic small-world networks, however, generated efficiency levels that during the whole course of development exceeded the levels in non-homeostatic small-world networks (Figure 11A). Remarkably, at the time when average activities reached the homeostatic set-point ϵ, efficiency levels of homeostatic networks further increased and almost reached the levels in random networks (Figure 11B). Consequently, favoring more distant connections in combination with homeostasis in electrical activity led to a more efficient network topology than achieved without homeostasis.

## 4. Discussion

We have shown that network formation favoring short-range over long-range connections produced networks with a pronounced small-world structure. Networks with homeostasis in electrical activity developed a weaker small-world structure in favor of more efficient wiring of connections. Global efficiency particularly increased when network activity reached the homeostatic set-point. Increased global efficiency was caused by the fact that homeostasis favored longer-ranged connections, which affected clustering as well as characteristic path length. Thus, network topology continued to change even after the network had reached a homeostatic equilibrium in electrical activity.

Adding more long-range connections to a small-world network makes the network more efficient but also more random. This is apparent in our simulations, too, by a decreasing clustering coefficient and a decreasing characteristic path length. Nevertheless, the small-world property of the networks was preserved throughout the whole course of development, and the decrease in clustering coefficient was slowing down over time. However, networks would most likely turn into random networks if rewiring continued indefinitely. Consequently, there seems to be a trade-off in network development between high clustering and strong small-worldness on the one hand and more randomness and higher efficiency on the other hand. The latter particularly arises when networks continue to rewire their circuitry when they are in a homeostatic equilibrium.

With additional long-range connections betweenness centrality decreases. Networks with low betweenness centrality are more robust against lesions because all nodes are equally important. By contrast, networks with high betweenness centrality are very vulnerable to lesions. If neurons that are part of many shortest paths are lost, the characteristic path length will immediately increase, with a significant impact on information transmission. It is remarkable that a self-organizing process that forms networks by striving toward homeostasis in electrical activity as a side effect produces topologies with lower betweenness centrality that contribute to a higher robustness against lesions.

Key to the homeostasis-driven change in topology is the increasing Euclidean length of connections. Since we did not observe an increase in connection length in networks without homeostasis, we concluded that the increase in connection lengths was caused by homeostasis in electrical activity. To create networks without homeostasis, we took the number of synapses from the homeostatic network at every update in connectivity and distributed them randomly under the same kernel in the non-homeostatic network. There are, in fact, other ways to create networks without homeostasis. We additionally built non-homeostatic networks by initially giving all model neurons a fixed number of vacant synaptic elements and then running updates in connectivity until no more synapses could be formed. Also in this scenario we did not see an increase in connection length. In a third scenario, we added a few vacant synaptic elements to all neurons before every update in connectivity. No matter how few vacant elements we added and how long we ran the network, we did not obtain more long-range connections than expected from the kernel. We only observed more long-range connections when we slowly added vacant elements at a few randomly selected neurons at a time (i.e., spatial sparseness in the formation of synaptic elements). From these observations we concluded that the contribution of homeostasis is not only to limit the number of available vacant synaptic elements but also to generate a certain sparseness in the formation of vacant elements. In fact, as long as the neurons were far away from the homeostatic set-point, synaptic element formation was not sparse at all as all neurons added elements roughly at the same time at equal rates. Only when the neurons approached the homeostatic set-point did sparseness in synaptic element formation arise. Homeostasis creates this sparseness because balancing the activity of one neuron immediately affects the activity in other neurons, which in turn may be driven further away from the homeostatic set-point and then start forming new vacant elements. The presence of inhibitory neurons would further reinforce this process.

Homeostasis in electrical activity is one way by which a local process can give rise to a change in global topology. Another comparable mechanisms was provided by Kaiser et al. (2009). In their model study, they showed that a simple axonal growth process can generate the experimentally observed exponential decrease in number of connections with increasing connection length. As a result of the growth process, most connections become short-range, although long-range connections also arise, but in lower numbers. The idea of their model is that axons grow out until they hit a postsynaptic target. The capacity of model neurons to receive connections is limited, and if nearby target neurons are completely occupied by incoming connections, axons continue to grow out until they hit a vacant target. Hence, Euclidean connection length increases over developmental time. In this model, the spatial growth process in combination with a hard boundary on the number of incoming synapses per model neuron generates the increase in connection lengths.

There are striking similarities between the topology of our model networks and that of developing dissociated cell cultures. It is well known that cultured neuronal networks can form small-world topology (Bettencourt et al., 2007; Yu et al., 2008; Gerhard et al., 2011). Downes et al. (2012) reported an increase in clustering coefficient of dissociated cell cultures between 14 days *in vitro* (DIV) and 28 DIV and a subsequent drop until 35 DIV, a course of development that is comparable to the course of development in our model networks with homeostasis. Between 14 and 35 DIV, the mean shortest path length did not change significantly, only showing a slight drop around 28 DIV. Consequently, small-worldness reached its maximum around 28 DIV. Moreover, the experimental data indicated the presence of longer synaptic connections from 28 DIV onwards that had not been not present at 21 DIV. From our previous studies we know that dissociated cell cultures reach homeostasis around this time (Tetzlaff et al., 2010). Therefore, we may hypothesize that the increase in connection length in dissociated cell cultures may be due to neurons reaching a homeostatic equilibrium in electrical activity. Other synaptic plasticity mechanisms not currently incorporated in our model may of course also have contributed to network formation in developing cell cultures.

On a macroscopic scale, functional imaging data reveal a development of small-world topology that also has interesting similarities with the self-organizing network formation in our model. The infant human brain has small-world properties already at the third post-natal week (Fransson et al., 2011). During the following 2 years, network topology undergoes a significant refinement: brain networks increase their small-worldness, global efficiency and number of long-distance connections (Gao et al., 2011). Although our network model shows only an initial transient increase in small-worldness, it may offer a simple explanation for the sudden increase in number of long-range connections and the associated increase in global efficiency. Could it be that even in the human brain, neurons establishing a homeostatic equilibrium in electrical activity produce—as an emergent property of the homeostatic growth process—more long-range connections? Remarkably, the increase in number of long-range connections occurs not during a genetically-encoded formation of an initial embryonic layout of projections but during the post-natal critical period (Gao et al., 2011), during which neurons are highly sensitive to afferent input. In general, the importance of critical periods is to balance excitatory and inhibitory circuits and to establish homeostasis in neuronal electrical activity (Hensch, 2005; Butz et al., 2009b). Considering that long-range connections arise in local as well as global networks, our study raises the interesting hypothesis that homeostasis in electrical activity may be the driving force for the formation of long-range connections on both a microscopic and a macroscopic scale.

Homeostasis in electrical activity is a ubiquitous principle in the nervous system (Wolff and Wagner, 1983; Ramakers et al., 1991; Abbott and Nelson, 2000) and a variety of plasticity mechanisms can act homeostatically. Scaling of synaptic strengths (Turrigiano, 1999), for example, has been reported as a mechanism acting at existing synapses to stabilize postsynaptic firing in cortical, hippocampal and spinal cord networks (Lissin et al., 1998; O'Brien et al., 1998; Turrigiano and Nelson, 1998). Even in the mature brain, not only the strength of synapses but also the formation of new synapses can contribute to the stabilization of neuronal activity, for example after focal retinal lesions (Butz and van Ooyen, 2013). In developing dissociated cell cultures, we showed that homeostasis in electrical activity may be a precondition for the emergence of self-organized criticality in neuronal firing (Tetzlaff et al., 2010). In another study, we showed that homeostasis in electrical activity can regulate the synaptic embedding of newly formed neurons in the mature hippocampal dentate gyrus (adult neurogenesis) and can account for the experimentally observed counter-intuitive inverse relationship between cell proliferation rate and synaptogenesis (Butz et al., 2008).

In summary, we conclude that homeostatic regulation of electrical activity together with simple distance-dependent formation of connections is capable of creating, in a self-organizing manner, neuronal networks that are more robust and more efficient than networks grown without homeostatic regulation. Strikingly, the growth process revealed features of developing topologies that are also observed in dissociated cell cultures and infant human brains. The formation of network topology by a self-organizing, local growth process may also be relevant for automatically generating the connectivity structure of large-scale neuronal networks (Potjans and Diesmann, 2014) that are currently studied in enterprizes such as the Human Brain Project (www.humanbrainproject.eu).

## Funding

This work was partly funded by the Helmholtz Association through the Helmholtz Portfolio Theme “Supercomputing and Modeling for the Human Brain.” Markus Butz and Ines D. Steenbuck were also partly supported by the NETFORM project (grant number 635.100.017, awarded to Arjen van Ooyen) of the Computational Life Sciences program of the Netherlands Organization for Scientific Research (NWO) and by a starting grant awarded to Markus Butz from the collaborative research center (SFB 874) funded by the German Research Foundation (Deutsche Forschungsgemeinschaft, DFG).

### Conflict of interest statement

The authors declare that the research was conducted in the absence of any commercial or financial relationships that could be construed as a potential conflict of interest.
